# Loneliness in adolescence: prevalence, developmental contexts, and interventions

**DOI:** 10.3389/fpsyt.2025.1696981

**Published:** 2025-11-07

**Authors:** Lily Verity, Lauren Burke, Pamela Qualter

**Affiliations:** Manchester Institute of Education, University of Manchester, Manchester, United Kingdom

**Keywords:** adolescence, loneliness, identity, peer relationships, social-emotional learning, interventions, mental health

## Abstract

Loneliness, though often mistaken for social isolation, is a distinct experience that affects individuals across the lifespan and is especially salient during adolescence. This developmental stage involves profound changes in identity, cognition, and social relationships, which can heighten vulnerability to loneliness. Drawing on global research, this paper highlights the widespread prevalence of adolescent loneliness, with variation shaped by cultural and societal factors. We explore how developmental transitions, shifting relational dynamics, and broader social environments contribute to experiences of disconnection, and consider the implications for mental and physical health. Loneliness in adolescence is associated with a range of negative outcomes, including depression, anxiety, social anhedonia, and increased health risks. Importantly, persistent loneliness may become maladaptive, reinforcing withdrawal through negative expectations and creating self-sustaining cycles of social disconnection. The review further examines intervention strategies aimed at reducing loneliness, including those that enhance social and emotional skills, strengthen supportive relationships, and address maladaptive cognitions. We argue that interventions must differentiate between transient and chronic loneliness, reflect the diversity of adolescent experiences, and prioritize co-production with young people to ensure developmental relevance and cultural sensitivity. Recognizing adolescence as a sensitive period for loneliness underscores the need for timely, evidence-based responses that equip young people to navigate disconnection and foster more supportive social environments.

## Introduction

Loneliness has historically been conflated with social isolation, presumed to affect primarily those who are physically apart from others. Yet, empirical research demonstrates that loneliness is not simply the product of isolation, nor is it confined to later life. It can occur even in the presence of others, such as during social gatherings, and is prevalent across the lifespan. Adolescence, in particular, is increasingly recognized as a developmental period characterized by heightened vulnerability to loneliness (1). This stage of life involves significant social, cognitive, and emotional transformations, including identity formation, changing relationship dynamics, and heightened sensitivity to peer evaluation. These transitions create unique challenges that can increase susceptibility to loneliness.

In this article, we examine loneliness exclusively in the context of adolescence. Drawing on global prevalence data, developmental theories, and empirical findings, we review how adolescent loneliness arises, the social and psychological mechanisms through which it is maintained, and the implications for health and wellbeing. We also discuss intervention strategies and identify critical research gaps. By situating loneliness within the developmental realities of adolescence, we emphasize the need for interventions that are developmentally sensitive, culturally responsive, and tailored to the nuanced experiences of young people.

## Prevalence of adolescent loneliness

Evidence from large-scale surveys and meta-analyses confirms that loneliness is a widespread issue among adolescents globally. Surkalim et al. (2), in a systematic review and meta-analysis, reported that loneliness among adolescents (ages 12–17 years) ranged from 9.2% in South-East Asia to 14.4% in the Eastern Mediterranean region. Igami et al. (3) with data from over 50 countries, showed that 11.7% of adolescents ages 13–17 years reported feeling lonely “most of the time” or “always” during the preceding year, with youth in Africa and the Eastern Mediterranean reporting the highest rates. Jefferson et al. (4) further demonstrated that cultural dimensions, including high power distance (a cultural context where inequalities in status and authority are accepted and hierarchy is respected) and low indulgence (a cultural context where enjoyment and personal freedom are restrained by strong social norms and self-control), were reliable correlates of adolescent loneliness across 75 countries.

Taken together, those findings underscore that loneliness is a global phenomenon in adolescence, but one with substantial cross-national variation. Cultural, social, and economic factors contribute to this variability, highlighting the importance of culturally sensitive approaches in both research and intervention. Measurement inconsistencies remain a challenge (5) given that standardized tools are not universally applied, and self-reported experiences of loneliness may be influenced by cultural norms regarding emotional disclosure (6).

## Developmental context of adolescence

### Identity formation

Adolescence is a critical period for identity development, a task central to Erikson (7) stage of identity versus role confusion. Marcia's (8) operationalization of Erikson’s theory highlights two processes: exploration (experimenting with roles, beliefs, and values) and commitment (deciding upon and adhering to specific identities). Exploration can be socially disruptive, leading to the dissolution of childhood friendships when values diverge. Moreover, the breadth of available identities can lead to ruminative exploration, characterized by persistent self-focus and indecision (9). Such rumination is strongly associated with loneliness (10), with adolescents in this state often withdrawing socially and experiencing heightened negative self-talk. Qualitative accounts reveal adolescents describing overthinking, not knowing where they fit, and feeling as if they are “on the outside looking in,” all hallmarks of identity-linked loneliness (11).

### Emotional and cognitive dimensions

The emotional landscape of loneliness in adolescence is more complex than in earlier developmental stages. While sadness, boredom, and frustration remain core emotions, adolescents additionally report apathy, grief, and hopelessness (12). Cognitively, loneliness in adolescence often involves self-blame and mistrust of social support; adolescents may discount reassurance from friends, believing themselves unworthy of that support (13). That cognitive style contributes to the persistence of loneliness, as even objectively supportive environments fail to mitigate feelings of disconnection (14).

## Changing roles of relationships

### Peer networks and friendships

Peers become increasingly central in adolescence, and peer acceptance strongly predicts loneliness. Being well-liked increases access to potential friendships, while rejection or exclusion heightens loneliness (14). The quality of friendships is critical: high-quality friendships characterized by support and validation protect against loneliness, whereas conflictual or ambivalent friendships increase vulnerability (15, 16). Meta-analyses demonstrate that negative friendship qualities often outweigh positive qualities in their associations with loneliness, suggesting that simply having friends does not suffice; the nature of these relationships is crucial (17).

Friendships during adolescence are multidimensional, comprising both positive and negative elements. Adolescents can experience simultaneous support and conflict within the same relationship. Importantly, perceptions of friendship quality differ between adolescents reporting loneliness and those adolescents who do not, with youth reporting loneliness perceiving their friendships as less supportive, even when objective indicators suggest otherwise (16).

### Romantic relationships

Romantic relationships typically emerge in mid-to-late adolescence and play an important role in shaping identity and social support. While romantic partnerships can provide intimacy and emotional closeness (18), they are often unstable, and frequent breakups can exacerbate loneliness and harm emotional wellbeing (19). For some adolescents, particularly those who place high social value on romantic involvement, the absence of a partner is associated with feelings of being unlovable and increased loneliness (20).

Sexual and gender minority youth face unique challenges in the romantic domain. Prejudice, discrimination, and difficulties related to disclosure (“coming out”) contribute to heightened loneliness among sexual minority adolescents (21). A comparative meta-analysis by Gorczynski and Fasoli (22) found that sexual minority individuals consistently report higher levels of loneliness than their heterosexual peers, across the lifespan. Minority stress frameworks view this through an intersectional lens by which interconnected socio-economic disparities, a sense of community, victimization and depressive symptoms relate experienced by sexual minorities result in higher levels of loneliness than non-minorities (23).

### Family relationships

Although adolescence is marked by increasing autonomy and a shift away from parental reliance, family relationships remain significant. Supportive family environments provide resilience against loneliness, while conflictual or emotionally unavailable family dynamics increase risk (24). Despite qualitative (13, 20), and quantitative (25) evidence that peer support is a much stronger link to loneliness compared to family support, parental support helps adolescents navigate social challenges; its absence exacerbates vulnerability, especially for those experiencing peer rejection or discrimination (20, 26).

## School and peer environments

Schools provide the primary context for peer interactions during adolescence. Peer networks within schools exert significant influence on loneliness. Asher and Paquette (27) demonstrated consistent associations between peer acceptance and reduced loneliness,

Peer rejection, whether objective or perceived, is a powerful determinant. Importantly, loneliness often reflects subjective experiences rather than objective social standing; adolescents may report loneliness despite not being rejected, or conversely, may not feel lonely despite rejection (28). Social norms further shape loneliness, as adolescents who perceive themselves as deviating from peer expectations—whether in appearance, interests, or socioeconomic status—report increased loneliness (29).

Bullying within school environments is also strongly linked to loneliness, with higher rates of bullying correlating with greater loneliness among students overall, not just victims (30). Thus, school-level policies on diversity, inclusion, and anti-bullying are crucial structural factors in mitigating loneliness (4).

## Social expectations and solitude

Adolescents experience strong social expectations to belong to peer groups. Nicolaisen and Thorsen (31) showed that older adolescents and young adults (18–29 years) reported higher expectations for companionship and intimacy within friendships compared to adults, with unmet expectations leading to loneliness. Social comparisons, particularly facilitated by social media, intensify these dynamics. Seeing peers engage in activities without them exacerbates feelings of exclusion.

At the same time, solitude plays a nuanced role. While solitude is often conflated with loneliness, adolescents sometimes describe “positive alone time,” characterized by reflection, self-discovery, and emotional regulation (32, 33). Nevertheless, social stigma against solitude can generate fear of exclusion, complicating adolescents’ ability to use solitude adaptively. Introverted adolescents may face particular challenges because peers often misinterpret preference for solitude as disinterest, further reinforcing loneliness (34). Indeed, recently, there have been public calls to change the narrative around solitude, so that time alone is viewed more positively and an essential part of wellbeing (35).

## Digital and societal influences

Digital technologies have reshaped adolescent social life, providing both opportunities and risks. Social media facilitates constant communication, but also exposes adolescents to cyberbullying, negative social comparison, and unrealistic expectations of relationships (36, 37). The quality and balance of online and offline interactions are likely to be critical: supportive digital interactions can reduce loneliness, whereas negative experiences amplify it.

Beyond digital contexts, structural inequalities also shape adolescent loneliness. Socioeconomic disadvantage, unsafe neighborhoods, and limited community resources are consistently associated with higher loneliness (38, 39). Discrimination, whether based on race, ethnicity, migration status, or minority identity, is a significant risk factor (6, 40). Adolescents from marginalized groups often experience compounded stress and reduced access to supportive relationships, placing them at heightened risk. Such approaches highlight the need for interventions at the structural level rather than the individual- or relationship- levels that have been prioritized by policymakers.

## Psychological and health outcomes

Loneliness in adolescence is strongly associated with poor mental health outcomes. A bidirectional relationship with depression has been established: loneliness predicts depressive symptoms, and depression increases vulnerability to loneliness (41). Anxiety and suicidal ideation are also more common among adolescents reporting loneliness (42). Qualitative accounts (20) describe loneliness as involving emptiness, demotivation, and a reduced capacity to enjoy social activity, phenomena linked to social anhedonia (SA). Unlike social anxiety, SA reflects disengagement due to diminished reward from social interaction, leading to withdrawal and further disconnection.

Prolonged loneliness triggers hypervigilance to social threat, biasing interpretations of social cues and reinforcing withdrawal, a process known as the “loneliness loop” (43, 44). This loop perpetuates loneliness, because adolescents’ negative expectations evoke behaviors that discourage others from engaging, thus confirming initial fears.

Loneliness also impacts physical health. It is linked to disrupted sleep, mild cognitive impairment, and increased risk for cardiovascular disease (45). Although many of these studies focus on adults, adolescent data suggest similar pathways, mediated by stress physiology and health behaviors (46).

## Interventions for adolescent loneliness

Recently, Goldman et al. (47) argued that efforts to address loneliness must be grounded in both theoretical insight and empirical evidence. Guided by those foundational theories, we argue that adolescent loneliness should be addressed through the development of social and emotional skills, the fostering of supportive peer networks, psychological intervention, and the reshaping of environments. Traditional interventions for adolescent loneliness have, however, aimed to increase opportunities for social contact, but those have shown limited success, with meta-analyses reporting only small effect sizes across the lifespan (48, 49). That limited impact is consistent with Social Needs Theory (50) and Cognitive Discrepancy Theory (51), which emphasize that loneliness arises not merely from the absence of contact, but from unmet emotional and social needs or from perceived discrepancies between desired and actual relationships. For adolescents, therefore, interventions must prioritize the quality and meaningfulness of peer relationships rather than the quantity of social encounters.

### Development of social and emotional skills

Social and Emotional Learning (SEL) programmes offer one promising universal approach in the management of adolescent loneliness (49). By fostering skills in emotional regulation, empathy, and relationship management, SEL aligns with Attachment Theory (52), promoting more secure relational patterns, and with the Evolutionary Theory of Loneliness (53), which emphasizes the need to reduce hypervigilance and mistrust in social contexts. Evidence suggests that SEL programmes improve coping and relational competence (54). Their cost-effectiveness and scalability make them particularly well-suited to school and community settings, consistent with socio-ecological perspectives that highlight the role of developmental environments.

### Fostering supportive peer networks

Targeted social support interventions, such as peer mentoring or befriending schemes, have demonstrated moderate effects on reducing loneliness in youth (55). Such approaches are theoretically grounded in Social Needs Theory, by addressing deficits in emotional or social support, and Attachment Theory, by offering new opportunities for trust and connection. Importantly, adolescents place greater value on peer support than on parental support (56, 57), underscoring the importance of facilitating peer-based, rather than solely adult-led, support structures.

### Psychological intervention

Psychological interventions also show considerable promise. Cognitive- behavioral therapy (CBT), interpersonal psychotherapy (IPT), and behavioral activation (BA), though traditionally designed for depression and anxiety, address maladaptive cognitions and social functioning difficulties central to loneliness, consistent with Cognitive Discrepancy Theory. Mindfulness-based approaches may reduce loneliness by dampening the heightened vigilance to social threat described in the Evolutionary Theory of Loneliness. Notably, digital delivery of CBT has proven effective for adults (58) and has been successfully adapted for adolescents (59). Such delivery modes not only enhance scalability but may also reduce stigma and increase acceptability among young people.

### Reshaping the environment

Efforts to reduce adolescent loneliness must extend beyond individual- or relationship- focused interventions to encompass the wider environments in which young people are embedded. Schools, as primary social and developmental contexts, are particularly influential. Research highlights that school climate, including the quality of teacher–student relationships, the extent of peer cooperation, and the presence or absence of discrimination, plays a critical role in shaping experiences of belonging and loneliness (4). Complex interventions that integrate social and emotional learning programmes, as noted above, within the curriculum, establish clear anti-bullying policies, and promote inclusive practices create conditions that support both social and academic flourishing. From a socio-ecological perspective (60), such environmental adaptations reflect the importance of addressing loneliness not only at the individual and relational levels, but also through community-level structures that foster meaningful connection and collective identity. Further, co-production with students is essential to ensure that any environmental changes reflect the lived realities of diverse adolescent populations, thereby enhancing their relevance and effectiveness. By embedding opportunities for connection into the fabric of the school, loneliness prevention becomes a sustained, context-sensitive component of adolescent development rather than an isolated or supplementary intervention.

Taken together, those insights suggest that adolescent loneliness interventions should be multilevel and theory informed. At the individual level, psychological therapies and digital tools can target maladaptive cognitions and reduce social threat sensitivity. At the relational level, mentoring and peer support schemes can strengthen meaningful connections. At the community level, SEL programmes and inclusive social initiatives can foster belonging. Finally, at the societal level, anti-stigma campaigns and culturally tailored approaches are needed to address structural barriers to connection. Such an integrated framework reflects the conclusion of Goldman et al. (47): loneliness in adolescence, as across the lifespan, requires interventions that move beyond increasing contact to fostering supportive, meaningful, and culturally sensitive relationships.

## Considerations for intervention design and evaluation

Future intervention research must distinguish between transient and prolonged (often referred to as chronic) loneliness because mechanisms and needs differ. Baseline measures of loneliness should be used to identify participants truly experiencing loneliness, avoiding mis-targeted interventions. Moreover, interventions must be inclusive of diverse adolescent populations, acknowledging cultural, socioeconomic, and identity-based differences in loneliness experiences. Co-production with adolescents is essential to ensure interventions are grounded in lived experience.

## Research gaps and future directions

Despite growing research, significant gaps remain in our understanding of loneliness among adolescents. First, most measures of loneliness were not co-developed with adolescents, raising concerns about developmental validity. Second, while qualitative research highlights nuanced experiences of loneliness, it remains underutilized relative to quantitative survey approaches. Incorporating adolescent voices through co-production can reveal mechanisms invisible to standardized measures, such as how social comparison and identity exploration intersect with loneliness. Third, more research is needed on minority and marginalized adolescents, who experience unique risks. Finally, intervention research must move beyond average effects to identify what works for whom, under what circumstances. This includes examining dosage, fidelity of implementation, and subgroup effects.

## Conclusion

Adolescence is a critical developmental stage characterized by heightened vulnerability to loneliness due to rapid social, emotional, and cognitive changes. Loneliness in adolescence is prevalent globally, with substantial cultural variation, and is associated with significant risks for mental and physical health. Peer networks, romantic and family relationships, social expectations, digital environments, and broader societal structures all play critical roles in shaping adolescent loneliness. While interventions such as SEL, psychological therapies, and social support programs show promise, further research is needed to refine those approaches and ensure they are developmentally appropriate, culturally responsive, and inclusive. Recognizing adolescence as a sensitive period for loneliness underscores the importance of timely prevention and intervention to mitigate long-term negative outcomes and support young people in building fulfilling social lives.
